# The American Year Book of Medicine and Surgery

**Published:** 1899-04

**Authors:** 


The American Year Book of Medicine and Surgery.—Being a yearly digest of scientific progress and authoritative opinion in all branches of medicine and surgery, drawn from journals, monographs and text-books of the leading American and foreign authors and investigators, collected and arranged with critical editorial comments by Samuel W. Abbott, M. D., John J. Abel, M. D., J. M. Baldy, M. D., Charles H. Burnett, M. D., Archibald Church, M. D., J. Chalmers DaCosta, M. D., W. A. Newman Dorland, M. D., Louis A. Duhring, M. D., D. L. Edsall, M. D., Virgil P. Gibney, M. D., Henry A. Griffin, M. D., John Guiteras, M. D., C. T. Hamann, M. D., Alfred Hand, Jr., M. D., Howard F. Hansell, M. D., Milton B. Hartzell, M. D., Barton Cooke Hirst, M. D., E. Fletcher Ingalls, M. D., Wyatt Johnston, M. D., W. W. Keen, M. D., Henry G. Ohls, M. D., Wendell Reber, M. D., David Reisman, M. D., Louis Starr, M. D., Alfred Stingle, M. D., G. N. Stewart, M. D., J. R. Tillinghast, Jr., M. D., J. Hilton Waterman, M. D., under the general editorial charge of George M. Gould, M. D. Illustrated. W. B. Sanders, 925 Walnut Street, Philadelphia. 1899.

This is not only a thorough and extensive resume of medical progress and thought, but, as will be seen by the title page, a thorough criticism by leading men in the profession of current medical topics. As suggested in reviewing the volume for 1898, one has to read this work to realize the vast extent of medical progress and activity. The physician who wants to know the very latest thing out, as well as the one who wishes to know the state of medical opinion on older subjects, has only to consult this volume. It will easily take the place, in all things except medical news, of any hundred medical journals published. The scope of the work is so great that space would not allow a summary, but some idea may be conveyed when it is stated that the work consists of 1100 pages, and that seventy pages of index are required to fully cover its contents. As a medical history of our times, if its publication is continued, it will prove an invaluable classic. J.

